# Supplementary material for: The histone methyltransferase Setd2 is indispensable for V(D)J recombination
Source: Nat Commun. 2019 Jul 26;10:3353. doi: 10.1038/s41467-019-11282-x (PMC6659703; doi:10.1038/s41467-019-11282-x)
Supplement: Supplementary file 1 — Supplementary Information [file 41467_2019_11282_MOESM1_ESM.pdf]

SUPPLEMENTARY INFORMATION

The histone methyltransferase Setd2 is indispensable for V(D)J recombination

by Zhongzhong Ji *et.al*

Supplementary Figure 1 | Setd2-deficient competitive bone marrow transplant recipients exhibit impaired reconstitution of T cells and B cells in the BM and peripheral blood. 2
Supplementary Figure 2 | H3K36me3 deposition was barely detected in Setd2 knockout thymocytes. 3
Supplementary Figure 3 | The rearrangement of TCRα was impaired in Setd2-deficient DP thymocytes 4
Supplementary Figure 4 | Expression of arranged TCRs abrogates the developmental block in Setd2-deficient thymocytes. 5
Supplementary Figure 5 | Loss of Setd2 impairs V(D)J rearrangement at the Igh gene locus and blocks B cell development at the pro-B stage 6
Supplementary Figure 6 | Conditional knockout in B cells caused impaired B cell development and aberrant V(D)J recombination at Igh loci. 7
Supplementary Figure 7 | Rag1 and H3K36me3 are enriched at Igh loci. 8
Supplementary Figure 8 | The expression and deposition of H3K4me3 are not reduced by *Setd2* knockout both in TCRβ and Igh genes loci. 9
Supplementary Figure 9 | *SETD2* is a recurrently mutated gene in human primary immunodeficiency patients. 10
Supplementary Figure 10 | Gating strategies used for flow cytometry data 11
Supplementary Table 1 : Complete Blood Count of Setd2 Knock out mice and control.12
Supplementary Table 2 : Antibodies 13
Supplementary Table 3 : Primers for qRT-PCR, genotyping and Knock-out SETD2 14
Supplementary Table 4 : Primers for ChIP assay 15
Supplementary Table 5 : Primers for Rearrangement assay 18
Supplementary Table 6 : Characteristics of Patients with *SETD2* mutations 19

Supplementary Figure 1

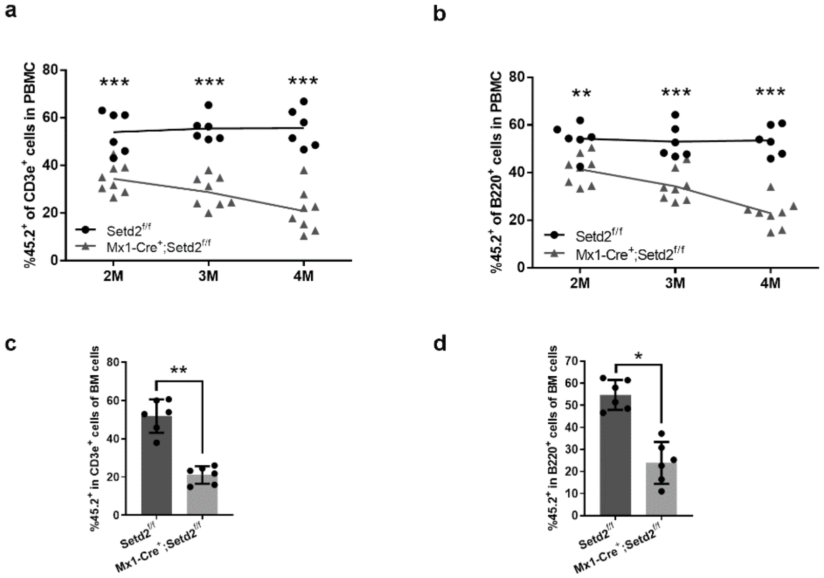

Supplementary Figure 1 | Setd2-deficient competitive bone marrow transplant recipients exhibit impaired reconstitution of T cells and B cells in the BM and peripheral blood.

(a-b) Chimerism of peripheral blood T and B cells in Setd2-deficient and wild-type bone marrow transplant recipients.

(c-d) Chimerism of bone marrow T and B cells in Setd2-deficient and wild-type bone marrow transplant recipients (n=14).

(The data are presented as the means ± SDs. *$p < 0.05$; **$p < 0.01$; ***$p < 0.001$)

Supplementary Figure 2

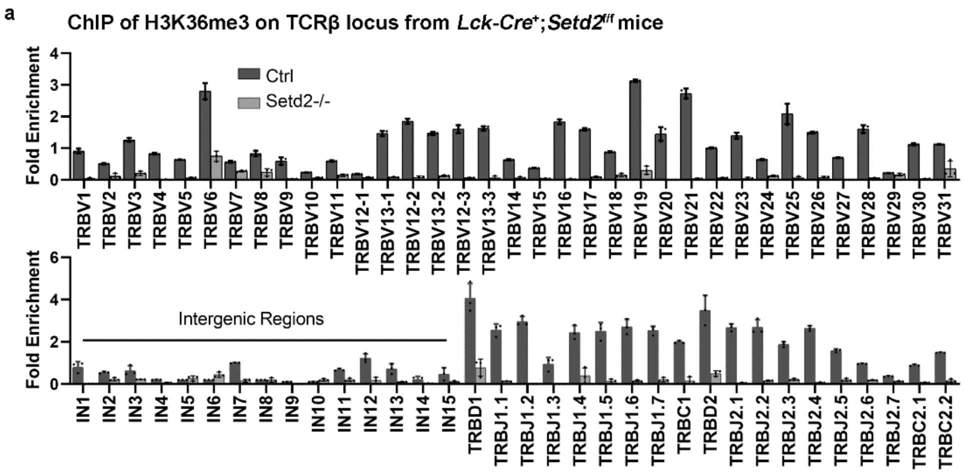

Supplementary Figure 2 | H3K36me3 deposition was barely detected in Setd2 knockout thymocytes.

(a) ChIP assays of H3K36me3 at the TCRβ locus from Lck-Cre^{+};*Setd2*$^{f/f}$ mice with specific deletion of *Setd2* in thymocytes; the kidney from the same mice was used as the non-rearrangement control.

Supplementary Figure 3

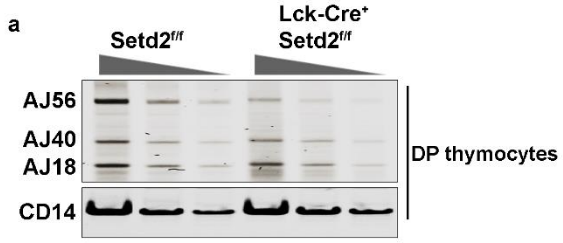

Supplementary Figure 3 | The rearrangement of TCRα was impaired in Setd2-deficient DP thymocytes

(a) Semi-quantitative PCR analysis for TCRα rearrangement. The TCRα gene segments were designed for multiplex PCR. The forward primer from TRAV15 and reverse primer from TRAJ56, TRAJ40 and TRAJ18 were used, and CD14 was used as loading control (double positive DP, $CD4^+CD8^+$).

Supplementary Figure 4

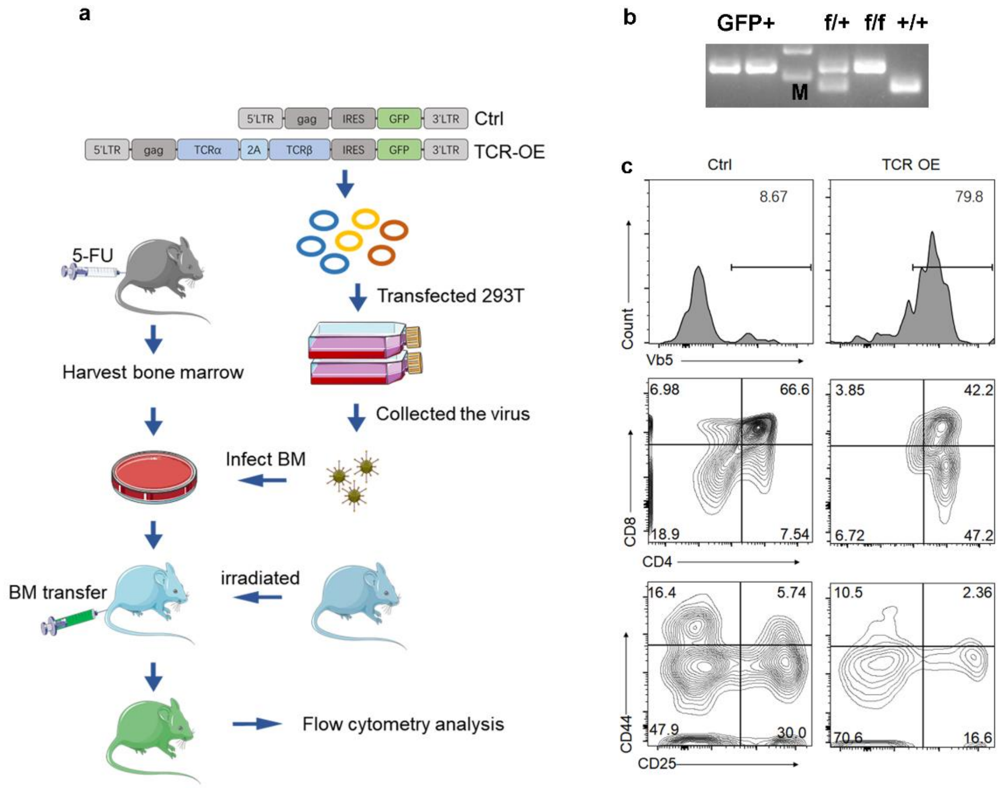

Supplementary Figure 4 | Expression of arranged TCRs abrogates the developmental block in Setd2-deficient thymocytes.

(a) Brief flow chart of the establishment of retrogenic mice for the expression of arranged TCRs in Setd2 knockout thymocytes.

(b) Genotyping using genomic DNA from GFP+ thymocytes from the recipients (retrogenic mice).

(c) Flow cytometric analysis of thymocytes from retrogenic mice transplanted with arranged TCR-expressing retrovirus or control virus.

Supplementary Figure 5

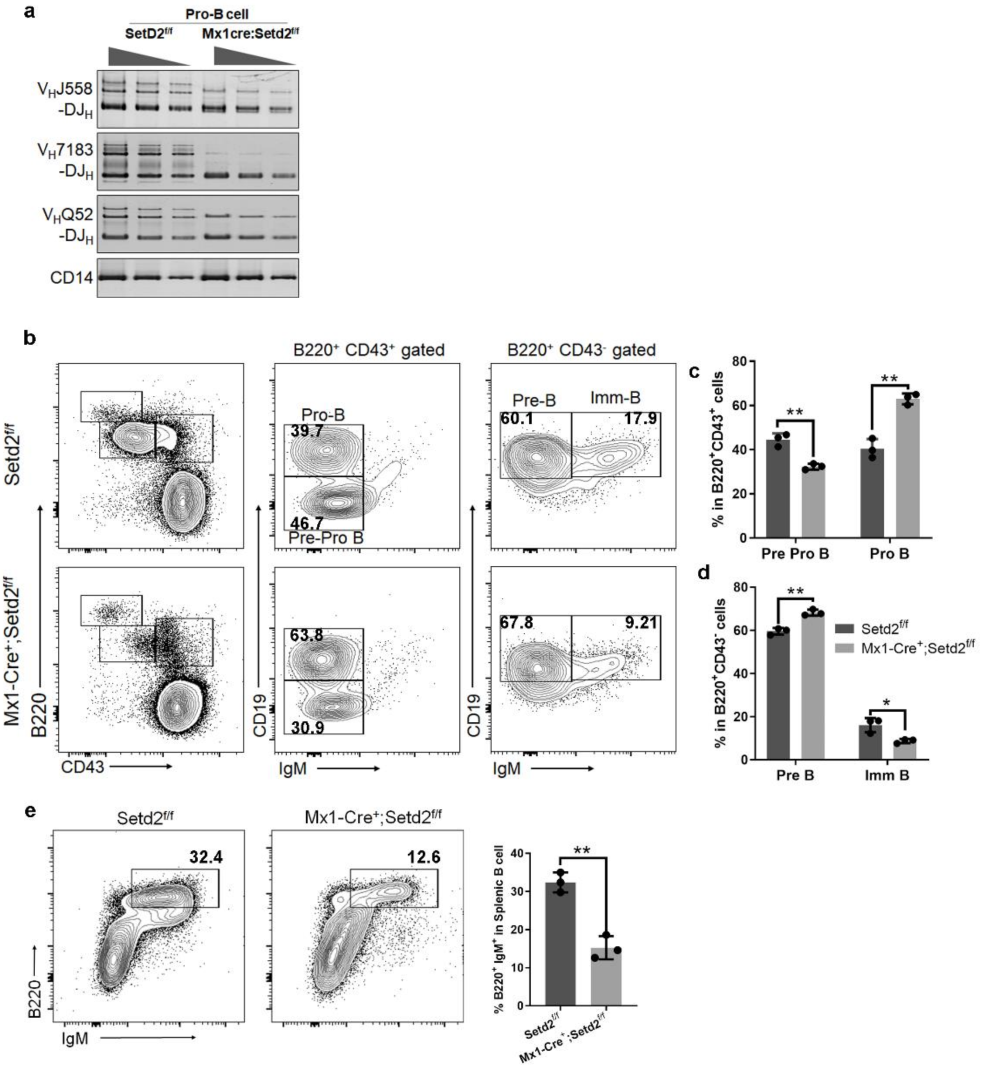

Supplementary Figure 5 | Loss of Setd2 impairs V(D)J rearrangement at the lgh gene locus and blocks B cell development at the pro-B stage

(a) Semi-quantitative PCR analysis of the indicated VH-to-DJH rearrangements in sorted pro-B ($B220^+CD43^+CD19^+IgM^-$) cells from the bone marrow of Mx1-Cre^+;*Setd2*$^{f/f}$ and control mice. V_HJ558-DJ_H and V_HQ52-DJ_H represent distal rearrangements, while V_H7183-DJ_H represents a proximal rearrangement. **(b-d)** Representative flow cytometry plots of B cell developmental profiles in the bone marrow of Mx1-Cre^+;*Setd2*$^{f/f}$ and control mice. Pre-pro-B (pre-pro B cells, $B220^{mid}CD43^+CD19^-IgM^-$), pro-B (pro-B cells, $B220^{mid}CD43^+CD19^+IgM^-$), pre-B (pre-B cells, $B220^{mid}CD43^-CD19^+IgM^-$), Imm-B (immature B cells, $B220^{mid}CD43^-CD19^+IgM^+$). **(c-d)** Histograms showing the frequencies of different subpopulations. **(e)** Flow cytometric analysis of splenic B cells in Mx1-Cre^+;*Setd2*$^{f/f}$ mice and control mice . (Data were collected at 8 weeks after the final pIpC injection. The data shown are presented as the means ± SDs. n=6, $^{**}p < 0.01$, $^{***}p < 0.001$).

Supplementary Figure 6

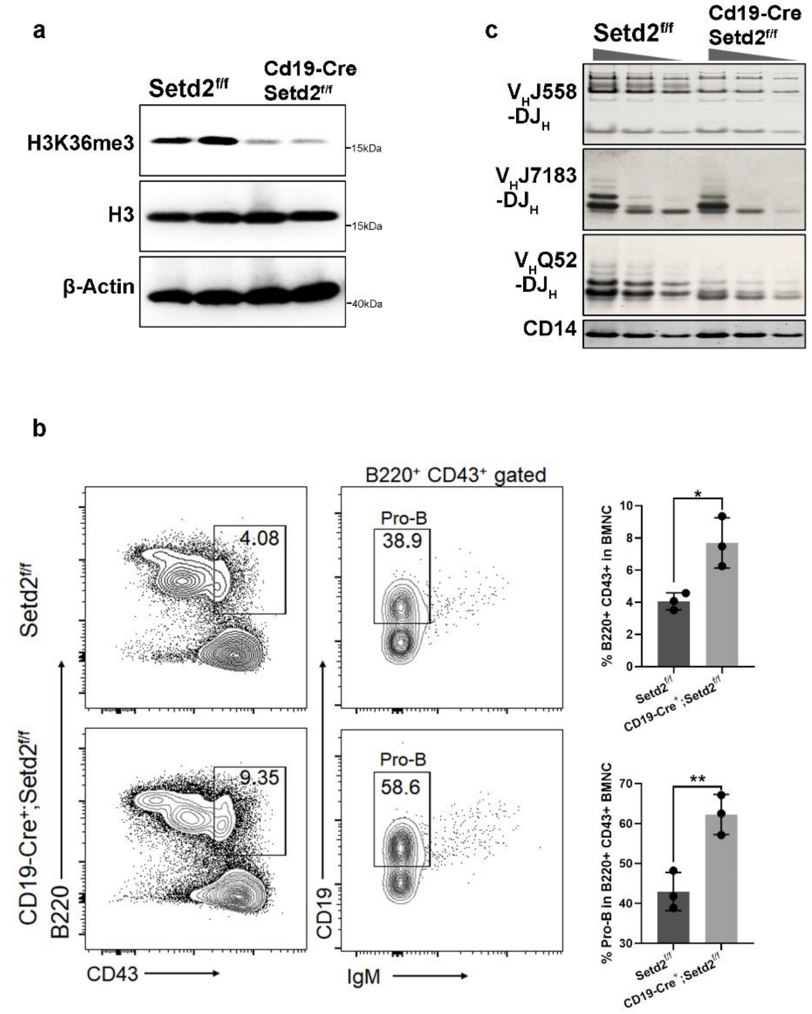

Supplementary Figure 6 | Conditional knockout in B cells caused impaired B cell development and aberrant V(D)J recombination at Igh loci.

(a) Immunoblotting of H3K36me3 in sorted B cells from Cd19-Cre^{+}; *Setd2*$^{f/f}$ and control bone marrow. H3 and β-actin were used as loading controls.

(b) Representative flow cytometric plots of pro-B (B220midCD43+CD19+IgM-) cells from Cd19-Cre^{+};*Setd2*$^{f/f}$ and control bone marrow mononuclear cells (BMNCs). The histograms show the frequencies of the B220+CD43+ and pro-B cell subpopulations in BMNCs (n=6).

(c) V(D)J rearrangement assay for pro-B cells from Cd19-Cre^{+};*Setd2*$^{f/f}$ and control mice. DNA was diluted to 3 concentrations ranging from 150 to 30 ng and subjected to 32 amplification cycles for V_H-to-D-J joint amplification or 24 cycles for CD14 amplification).

(The data are presented as the means ± SDs. *$p < 0.05$; **$p < 0.01$; ***$p < 0.001$)

Supplementary Figure 7

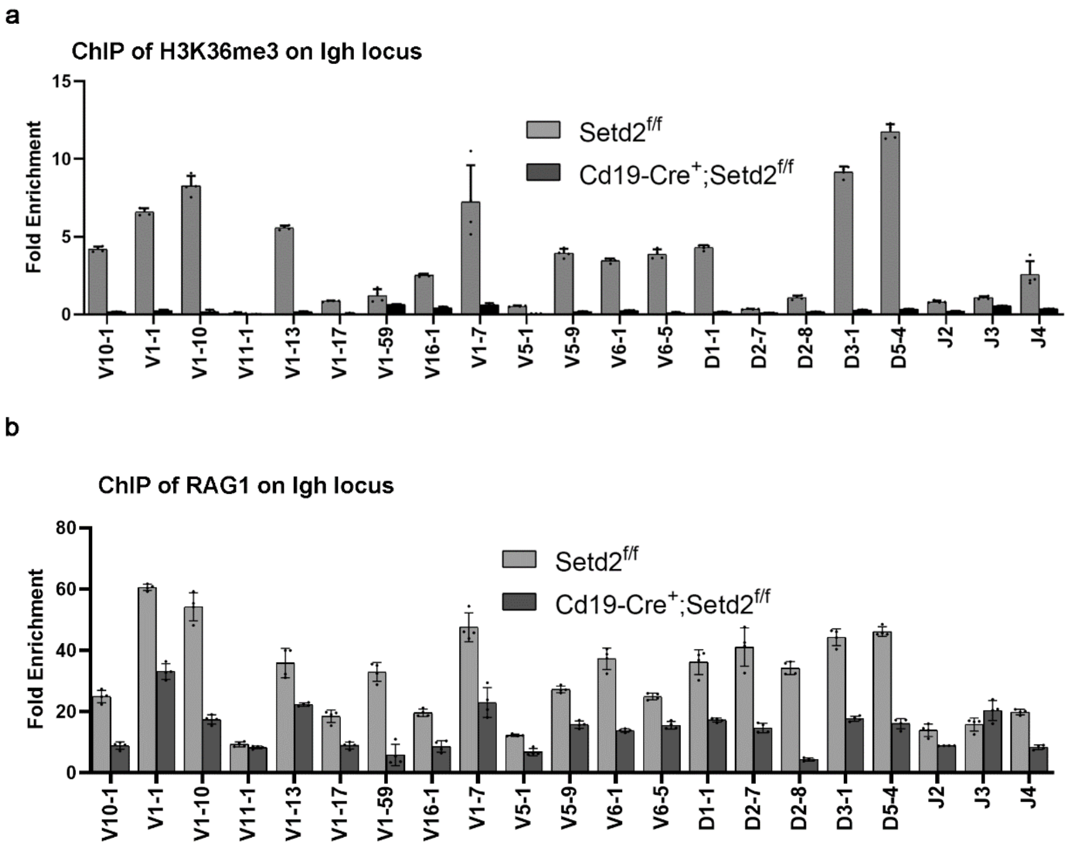

Supplementary Figure 7 | Rag1 and H3K36me3 are enriched at Igh loci.
(a) ChIP assay of H3K36me3 at selected segments of Igh loci in B220+CD19+ B cells from *Cd19*-Cre^{+}; *Setd2*$^{f/f}$ mice and control mice.
(b) ChIP assay of Rag1 at selected segments of Igh loci in B220+CD19+ B cells from *Cd19*-Cre^{+}; *Setd2*$^{f/f}$ mice and control mice.

Supplementary Figure 8

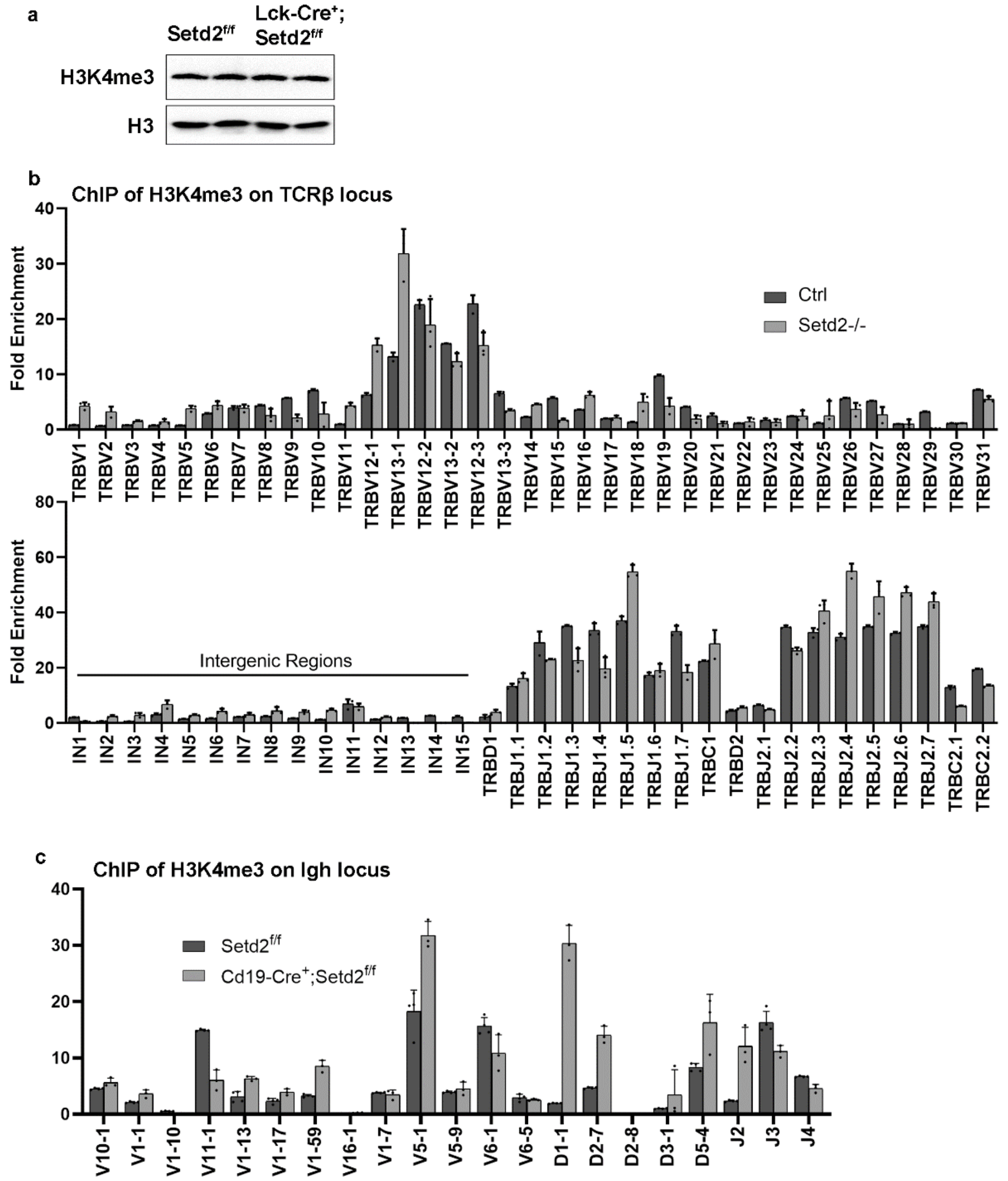

Supplementary Figure 8 | The expression and deposition of H3K4me3 are not reduced by *Setd2* knockout both in TCRβ and Igh genes loci.

(a) Immunoblotting of H3K4me3 in sorted (CD3e+) T cells from Setd2-deficient mice. H3 was used as the loading control.

(b) ChIP assay of H3K4me3 at the TCRβ locus in DN thymocytes from Lck-Cre+;*Setd2*$^{f/f}$ mice and control mice.

(c) ChIP assay of H3K4me3 at the Igh locus of B220+CD19+ B cells from Cd19-Cre+;*Setd2*$^{f/f}$ mice and control mice.

Supplementary Figure 9

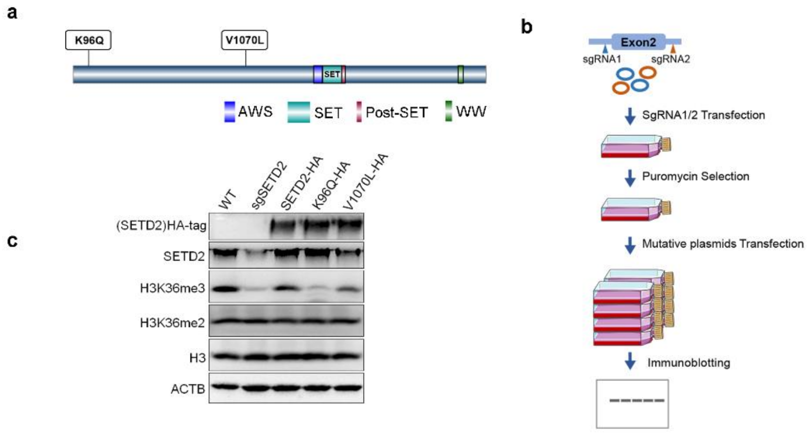

Supplementary Figure 9 | *SETD2* is a recurrently mutated gene in human primary immunodeficiency patients.

(a) Schematic diagram of *SETD2* mutations in human primary immunodeficiency patients.

(b) Procedure for the functional reconstitution assays of *SETD2* mutations in 293T cells.

(c) Immunoblotting of SETD2 and H3K36me3 in 293T cells with CRISPR/Cas9-mediated *SETD2* knockout transfected with plasmids expressing wild-type or mutated SETD2.

Supplementary Figure 10

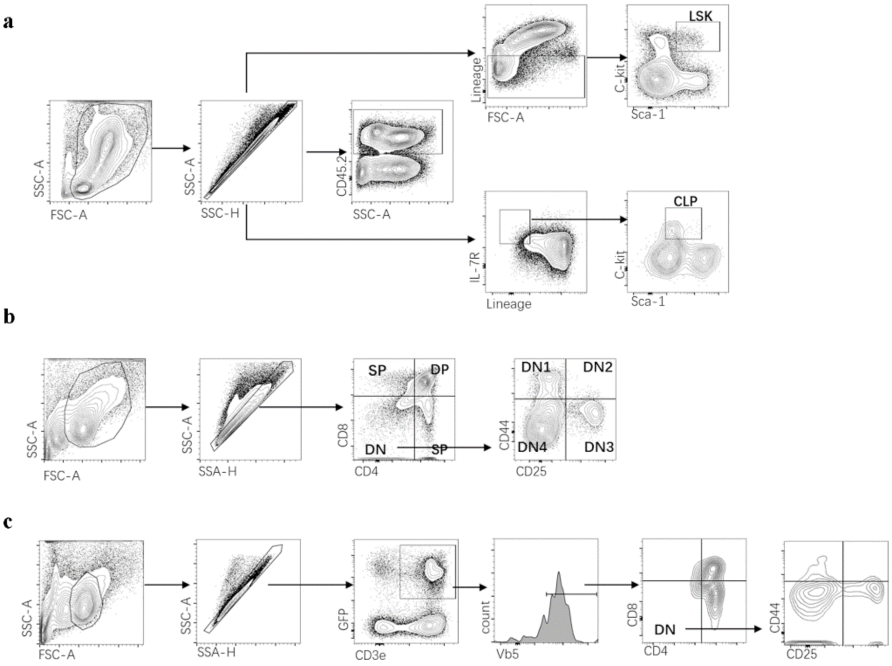

Supplementary Figure 10 | Gating strategies used for flow cytometry data.
(a) Gating strategy for identifying Hematopoietic stem cell enriched population (Lineage^{-}c-kit^{+} Sca-1^{+}, LSK) and common lymphoid progenitors (Lineage^{-} IL7R^{+} Sca-1^{mid} c-kit^{+}, CLP). **(b)** Gating strategy for identifying single positive T cells (CD4^{-} CD8^{+} or CD4^{+}CD8^{-} ,SP), double positive T cells(CD4^{+}CD8^{+} ,SP), double negative T cells(CD4^{-}CD8^{-} ,DP), DN1(CD4^{-}CD8^{-} CD44^{+}CD25^{-}), DN2(CD4^{-}CD8^{-} CD44^{+}CD25^{+}), DN3(CD4^{-}CD8^{-} CD44^{-}CD25^{+}), DN4(CD4^{-}CD8^{-} CD44^{-}CD25^{-}).**(c)** Gating strategy for analyzing the retrogenic TCR mice, TCR(Vb5) co-express with GFP.

Supplementary Table 1: Complete Blood Count of Setd2 Knock out mice and control.

	Setd2$^{f/f}$	*Mx1-Cre*$^{+}$*;Setd2*$^{f/f}$	
WBC(10^9/L)	5.580±0.482	2.453±0.193	***
PBL(10^9/L)	3.833±0.298	1.547±0.152	***
MO (10^9/L)	0.215±0.033	0.156±0.037	ns
RBC (10^12/L)	5.483±0.279	4.898±0.321	ns
Hb (g/L)	142.429±5.355	129.250±7.974	ns
HCT (%)	21.860±0.224	19.733±2.532	ns
MCV (f1)	39.267±2.937	38.918±2.172	ns
MCH (pg)	24.980±0.315	27.325±1.087	**
MCHC (g/L)	669.333±45.829	681.000±26.184	ns
RDW (%)	44.275±5.421	34.317±3.230	**
PLT (10^9/L)	374.750±38.491	478.462±35.674	**
MPV (f1)	5.100±0.141	4.229±0.243	***

Complete blood count of the peripheral blood of *Mx1-Cre*$^{+}$*;Setd2*$^{f/f}$ and control mice after 8 weeks treated with three doses of PIPC. (The data are presented as the means ± SDs. *$p < 0.05$; **$p < 0.01$; ***$p < 0.001$; ns, not significant)

Supplementary Table 2: Antibodies

Antibodies used for flow cytometry

ID	Clone or Catalog No.	Vol./10^6 cells	Brand
Mouse Lineage Panel	Biotin label	4 µL	BD® Bioscience
Sca-1	D7	1.5 µL	eBioscience™
C-Kit	2B8	1.5 µL	eBioscience™
CD3e	145-2C11	3 µL	BD® Bioscience
CD4	RM4-5	3 µL	BD® Bioscience
CD8	53-6.7	3 µL	eBioscience™
CD44	IM7	2 µL	eBioscience™
CD25	PC61.5	2 µL	eBioscience™
CD45.2	104	3 µL	eBioscience™
B220	RA3-6B2	3 µL	eBioscience™
CD19	1D3	3 µL	BD® Bioscience
IgM	II/41	2 µL	eBioscience™
IL-7R	SB/199	1.5 µL	eBioscience™
FcγR	93	1.5 µL	eBioscience™
TCR-Vb5.1	MR9-4	3 µL	Biolegend
Streptavidin	V450-Streptavidin	5 µL	BD® Bioscience

Antibodies used for IHC and western blotting

ID	Clone or Catalog No.	Dilution	Brand
SETD2	#23486	1:1000	Cell Signaling Technology
SETD2	#3194	1:1000	Abclonal
HA-tag	#3724s	1:1000	Cell Signaling Technology
H3K36me3	ab9050	1:1000	Abcam
H3K36me2	ab3594	1:1000	Abcam
H3K4me3	#9751	1:1000	Cell Signaling Technology
H3	ab1791	1:3000	Abcam
RAG1	#3968s	1:500	Cell Signaling Technology
RAG2	ab133609	1:500	Abcam
γH2A.X (p-S139)	ab26350	1:500	Abcam
ATM	ab78	1:500	Abcam
ATM (p-S1981)	ab81292	1:500	Abcam
β-Actin	#3700	1:3000	Cell Signaling Technology

Supplementary Table 3:Primers for qRT-PCR, genotyping and Knock-out SETD2

Primers for Real-time PCR

ID	Sense 5'-3'	Antisense 5'-3'
mSetd2	CATAGCTGTGAACCAAACTGTGA	TAATTCTGAGCCTGAAGGAACTA
mRag1	GGCTAGGGTCAGCAGCAAGGA	CACGGGATCAGCCAGAATGTGTCC
mRag2	CAGAACTTCAGGATGGGCTGTCTTT	TTTGAGTGAGGATTGCACTGGACAC
mNHEJ1	TCTGGAGAGACTCAGGCATCAA	AGCAGCCACAGGCTCATATTCA
mKu70	GGTGCCCCAGGAAGAGGA	TGTATGTGAAGCGGAGCTTTTG
mKu80	CAGCGCTTCAACAGCTTCCT	TCCACATCACCAGCTTCTTCA
mXrcc4	ATGATGGAAGCACCGATGAA	CTTCTCCTGTAGCGGCAACTC
mLig4	TGAGCTGCGGTTTCATGG	CTGCAGTTCGCCCTTGTCTA
mDna-pk	CCCGCCAATTTGTCAGTCT	TCCTCCTTTTCTCAGCATTGTC
mTAOK	ACAACAACCTAAGAGTCTGAAGTCT	TGGTGTAGTCTCCAGTAGGTGA
mDCLRE1C	CTGCACTCTGGAGGCAGAGT	CACTGCTCACGACTTGGGATCT
mMDC1	CAGCACCCAGAAGCTGTCCA	CACCTGGAGTCTGAAGGCTGA
mCHEK2	CCGGACTTACAGCAAGAAGCA	CTGTGATCCTCTATGTATACGATGT
mPRKDC	AATTGCATTAGTGCTGTGGTGCA	GGTTATCAGACCTAGACTCAACAT
mActin	GTGGCATCCATGAAACTACA	CTCATCGTACTCCTGCTTGC

Primers for genotyping

ID	Sense 5'-3'	Antisense 5'-3'
Setd2	TGCTGAGGAAACACCATCCC	GATCTCTGAAGGCAGCAGCA
Mx1-Cre	TTTCGTTTCTGAGCTCCTTTC	ATTGCCCCTGTTTCACTATC
Lck-Cre	TTACCGGTCGATGCAACGAGTGA	AGCTGGCCCAAATGTTGCTGGATA
Cd19-cre	GCGGTCTGGCAGTAAAAACTATC	GTGAAACAGCATTGCTGTCACTT

Primers for knock out SETD2

ID	Sense 5'-3'	Antisense 5'-3'
sgSETD2-1	caccgATGGTAACTTATCCCAGTGA	aaacTCACTGGGATAAGTTACCATC
sgSETD2-2	caccgAGAAATCAGTCGGCAGGACA	aaacTGTCCTGCCGACTGATTTCTC

Supplementary Table 4:Primers for ChIP[1] (TCRβ loci)

Primer ID	**Sense 5'-3'**	**Antisense 5'-3'**
TRBV1	CCACAGTGACTTTGCTGGAGC	CTCCGCAGAGTGAACAGCCACTGT
TRBV2	TGGTGGCAGGTGAGTCCTAGT	TCCCTGTGACTGCCACCAGAT
TRBV3	ATGGATATCTGGCTTCTAGGTTGGAT	ATTTGATGACTTGGGATCTGTAAGA
TRBV4	TGGCCTTCTGCCTCTTGGGAAT	CTGTGTGACATGATAGTTTGGAGT
TRBV5	CAGGTGAGACTTGGATTCCACA	CCCAGGATTAGATATCTTGGTGA
TRBV6	CCAGAGGAAATATGGAATCTGGA	AGAATGAAGCCAGAAACTCCACA
TRBV7	GTCTCCTGGGAGCAGATGAATCA	GGTTTCAGGTTACATGACTCTGGAA
TRBV8	TGCAGGCAGGTGAATTCTGTGCA	GAATGATACCAGCACTCACTGGA
TRBV9	GACCTCACTAGTGCTGAATTCT	GCCTCATCTTGAGTTCTGTCAGTA
TRBV10	TGGAATCACCCAGACACCTAGA	CAGTGCCTACTGAGTAATGGATCA
TRBV11	CTTCTGATATGTGGCCTCTCACTT	GGTGATCCTATGAGTAGGAGACT
TRBV12-1	GTCTAACACTGTCCTCGCTGA	GGACAACCCCAGAATCTGCTGAA
TRBV13-1F	GAGCCTCCTGTGTACAAGTGA	CTAGGGCTTTGGGTGACTGCA
TRBV12-2	CCTGGGAACAAGTAAGTCCAGA	TGGAGACTGGACAACCCCAGAA
TRBV13-2	CCAGTCTCCTGTGTTCAAGTGA	GGTGACTGCAGCCTCCATGT
TRBV12-3	CCTGGGAACAAGTGTGTCCTGA	GGACAACCCCAGAATTTGCTGA
TRBV13-3	TTGATTCTCCTGTGTGCAAGTGA	GACTTTGGGTGACTGCAGCCT
TRBV14	TCCTCTGCCCTCAATCTGCCA	GTGACTCCAGCTTCAGACAGTA
TRBV15	TCCAGACCCTCTGTTGTGTGAT	GGTGTCTGGGTAACTCCAGCA
TRBV16	AAGCCACTGCCTCATCTTGCCA	TGACACCAGCATTTGTTGGTTCTA
TRBV17	TGGACCATCCATGGATCCTAGA	GTTCTGCTTAACCGTAGTATCCA
TRBV18	ATGTCTCAGGAATGCTGGTGTCA	CAGCATTCCTGGCCTCTCTAA
TRBV19	GTAACCCTTTGTCTCCTTACTGTA	GCCACCATCACCATGTGTGGT
TRBV20	GTGGTGTGAAGTCAACACTGAAGA	CAAGGTATAGCATGGCTCATACCA
TRBV21	TCAGCACTCTCTATGAATCAACCA	CCAGAGTCCATGGAGCCTGT
TRBV22	TATCTCCTAGTAGCAGGTGAGTC	TTGGCAATCCAAAGAGCCTAGAA
TRBV23	CGGCTCATTTGCTATGTAGCAC	GCTTCTGTGTAACTGCAGCATC
TRBV24	CTAGTGTGATCATGGGTGCAAGA	TGGAGTCTGGGTTACTCCAGCAA
TRBV25	CTTGATACAAAGTGTGAGAACCTCA	CTGAGGACTCATCTGCTCCAGT
TRBV26	TGTCTCCTGGGTGCAAGTAAGT	GTTGGCAATCTCCAGGTCAAAGA
TRBV27	TAATCTGGGAGCAGGTAAGATGCA	GATAATAGCATCCAATAGGCCTGCA
TRBV28	CTATGAACAGGTGAGTTCTGAAACA	GGGCTTCTGTGGAGACTACGA
TRBV29	CTGTGTTTCCTAGGAACAGGTGA	TGTCCACAAGGCCTGTGGTGAA
TRBV30	AGGAGCCTTGTCCTGAGCTGA	TGTCTCCCTCATCCTCTAACTAGT
TRBV31	GAGACTCCAGGCACAGAGGTA	GTGGTGCAACTGAACCTCTCA
TRBD1	GTTTCTTCCAGCCCTCAA	CCCCATTCTTCTCCACAA
TRBJ1.1	CTCCTCATCCTATGGCACTGT	ATCTTACCTACAACTGTGAGTCT
TRBJ1.2	TGCAAACTCCGACTACACCTTC	ATGAGCAGCATCCTTCCTCTGA
TRBJ1.3	CTGTGTTCTGGAAATACGCTCTA	TCAGAGAGAAACTTCACCCAGTA
TRBJ1.4	CGGTCATGGAACCAAGCTGTCT	CGTGGCTCTAGGTTTACAACACA
TRBJ1.5	CTCATGTTGTACTGTGTAACAACCA	CAGTTTGGTCCCATAGTTTACCTA

TRBJ1.6	CCACCTGCAGCTGTGTTCCTA	CTCACTGTGACAGGTATGGG
TRBJ1.7	CCTGTGTTGGATGACCATGGT	AGGGACGACTCTGTCTTACCTTA
TRBC1	AGGATCTGAGAAATGTGACTCCA	GACCTCCTTGCCATTCAC
TRBD2	CCAAGCTCCTCCCCTCTTTAGA	AGCACCTCTTCCAGTTGAATCATT
TRBJ2.1	CTGCTGTGTAACTATGCTGAGCA	TATGGCCTCTGCCTTCTTACCTA
TRBJ2.2	AGCATTTCCAGGACTGTGCAAACA	CACTGTCAGCTTTGAGCCTTCA
TRBJ2.3	GCCTGGAGGCTGTGAGTGCA	CCAACTTACCGAGAACAGTCAGT
TRBJ2.4	TGTACCAAGAGGCTGTGAGTCA	CAGCTTACCTAGCACCGATAGT
TRBJ2.5	CTGTGAACCAAGACACCCAGTA	CCTAACACGAGGAGCCGAGT
TRBJ2.6	ACTGATTGGCAGCCGATTGAACA	GCAGTTGCCAAGAAGCTCGCA
TRBJ2.7	CCTCTGTGCTCCTATGAACAGTA	TGGAAGCGAGAGATGTGAATCTTA
TRBC2.1	CCAGAGGATCTGAGAAATGTGAC	AGCTCAGCTCCACGTGGTCA
TRBC2.2	CCTGTCAACAGCATCCTATCATCA	GTCCATCCTACCATTCTTACCAT
IN1	TGCCTGCTCCATCTGGAGCA	TAGGAAGAAGGAAGGACTGGAC
IN2	GACTAGGCCATCCTCTGCTACA	GACTTGCTAAGTGTGAACTATGACA
IN3	CTTTAAGTCTGATGCTGCACTC	AGGCGTGGACATTAACACATCTAC
IN4	CAGAACTCCAAATAGACTGGACCA	CTGGCTTTCATAGTCTCTGGTGA
IN5	AGCACGTAAGGAAGCACAGAAGA	GTGGGAGGGCAGATTGTGAGA
IN6	CTTCAGAGGCTCTTAGAACATTGA	TCTGAAGAACTCATTGAGTCCTGA
IN7	GACCATGGAGTGGCACCATC	GAAGACTGACTTCGAGGCAACTA
IN8	GCAGACAGTACCCACTGGTCA	GGTGCTATACTTGTCAGGAGCA
IN9	GTGATATTGAATGTAAGTCACAGGA	CCCATGGTATTTCATACTCTATCA
IN10	CAGCTAGCTTCAGAGATTACCAGA	CTCTTTAGCAGTGCTCTCCTGT
IN11	AGCAAGCCGCACCTCTGGTA	CGACAAACCCTGAGCCAGTC
IN12	TACCTGGTGTTCGAACCAGACT	TGGAACTTGAGACTGCGGTAC
IN13	AAAGGATGGACCATCCAGAGACT	CCAGGCAGTGGCATAACCTCA
IN14	TCCACTCCTGATGTCCACATTC	GACAAACATGGTATGTACTCACTT
IN15	TCATCCACTGAATCACCAAGTAC	GTCTGAGAGATCTTAGGAGTCCA

Primers for ChIP (Igh loci)

Primer ID	**Sense 5'-3'**	**Antisense 5'-3'**
V10-1	GTCTGCATCTTCATCTGAGCTGA	ATCTTCAGTGTGAGCCTAGACA
V1-1	ACGTCACAGTGAGGATGTGACA	CTAGGCACATATCCTCCAGCAT
V1-10	CTACTCCATGAGAGTTTCTGACA	CTGACATCTGAAAACTCTGCAGTC
V1-13	TTCTGACACTCTCAGGATGTGG	CCAACACAGCCTACATGCAACT
V1-17	TAAGTCTTGTCAGTCTCAAGGCA	CAAGACACAGTGTTGTAACCACA
V1-59	CATACTACACACCATCCTGGCT	AACCCTGGAGGAGTAGCAAACT
V16-1	TGCTGGTCCTGAGCTCCTTGT	TCTGAGAAACGAAGACACGGCT
V1-7	TCATCAAGCCTACAGGTTAGTC	AGACACAGTGGTGCAACCACAT
V5-1	TTTGTGTTTGAGCTCACAGTAACA	CCGATTCACCATCTCCAGAGA
V6-1	CTTCCTACACAAGCCATGGGTA	GCAACATGTTATGGAGGTTTGT
V6-5	GTATCAATAGATAGTGGTGTCACC	TGTAGGTGTTCTGAGGTCCACTA
D1-1	CTAGACTCAGTTTTTGGAGCTCAA	CTACGGTAGTAGCTACCACAGT
D2-7	GCTGAAGAGTCTGCTGGGCATA	AGAACGTGTTACCTTACTTGGCA

D2-8	CTGTGGTAGTTACCATAGTAGAC	CTCTGGCCCCACCAGACAAT
D3-1	AAAGCCAGAAAGGGAATAGGTCT	CTGTCACAGTGGGCACAGCT
D5-4	CTGACTGGCTAAACACTGTAGA	CACAAGAGGTGGATTCTGTATGT
J2	GAGGTTGTAAGGACTCACCTGA	ACATTGTTAGGCTACATGGGTAGA
J3	CTGCAGAGACAGTGACCAGAGT	TGGAGCCCTAGCCAAGGATCA
J4	CATTCTTACCTGAGGAGACGGT	GTGTGACACCAGGAATTGGCAT

Supplementary Table 5:Primers for Rearrangement assay [2]

ID	5'-3'
TRBV3	GATTTTAGGACAGCAGATGGAGTTTC
TRBV12-2	CAGCAGATTCTCAGTCCAACAGTTT
TRBV30	CCGGCCAAACCTAACATTCTCAAC
TRBD1	GAGGAGCAGCTTATCTGGTGGTTT
TRBD2	GTAGGCACCTGTGGGGAAGAAACT
TRBJ1.7	AAGGGACGACTCTGTCTT
TRBJ2.7	TGAGAGCTGTCTCCTACTATCGATT
J_H3R	GTCTAGATTCTCACAAGAGTCCGATAGACCCTGG
V_HJ558	CGAGCTCTCCARCACAGCCTWCATGCARCTCARC
V_H7183	CGGTACCAAGAASAMCCTGTWCCTGCAAATGASC
V_HQ52	CGGTACCAGACTGARCATCASCAAGGACAAYTCC
TRAV15	TCCATCAGCCTTGTCATTTCA
TRAJ56	TGGTATAACACTCAGAACGGT
TRAJ40	TGGTACCTGCTCCAAAGACG
TRAJ18	CTGAACCTCTATCTACACAGTG
CD14-F	GCTCAAACTTTCA GAATCTAC
CD14-R	AGTCAGTTCGTGGA GGCCGGAAATC

Supplementary Table 6: Characteristics of Patients with *SETD2* mutations

Characteristic	Patient 1	Patient 2	Patient 3	Patient 4	Patient 5	Patient 6	Patient 7	Patient 8
Age	1 months	12 months	10 months	7 months	2 months	4 years	3 years	3 months
Sex	Male	Male	Female	Male	Female	Male	Male	Male
SETD2 mutation	V1070L	H1026Y	S1792N	K96Q	N1943S	H216D	A1382S	K308E
Clinical manifestations	Severe infection	Recurrent rush	Recurrent fever，High lever CRP	Skin infection with Streptococcus pneumoniae, liver function damage	Tongue ulcer, pulmonary infection	recurrent acidocytosis，pulmonary infection，	Recurrent infection，hyperacidophilia，lymphadenectasis	Infection resulting in sepsis
IgG (g/L)	3.04	ND	6.6	2	3.87	9.4	12.7	5.53
IgA (g/L)	0.08	ND	0.26	0.28	0.09	1.98	0.97	0.28
IgM (g/L)	0.3	ND	1.24	0.32	0.63	0.74	2.2 1	0.29
IgE (KU/L)	185	ND	24	2530	11	55789	193	10
CD3+T (/ul)	10	ND	ND	9161	2729	2233	3679	997
CD4+T (/ul)	4	ND	ND	7120	2200	1376	1924	739
CD8+T (/ul)	4	ND	ND	1785	450	664	1459	220
CD19+B (/ul)	847	ND	ND	1259	1057	618	1203	1100
CD16/56+NK (/ul)	42	ND	ND	1528	246	232	490	311
ND : No Data								

Supplementary Reference

1. Rupp, L. J., Chen, L., Krangel, M. S. & Bassing, C. H. Molecular Analysis of Mouse T Cell Receptor α and β Gene rearrearearrangement. **1323,** (2016).
2. Gärtner, F. *et al.* Immature thymocytes employ distinct signaling pathways for allelic exclusion versus differentiation and expansion. *Immunity* **10,** 537–546 (1999).
