# Supplementary material for: The histone methyltransferase Setd2 is indispensable for V(D)J recombination
Source: Nat Commun. 2019 Jul 26;10:3353. doi: 10.1038/s41467-019-11282-x (PMC6659703; doi:10.1038/s41467-019-11282-x)
Supplement: Supplementary file 3 — Reporting Summary [file 41467_2019_11282_MOESM3_ESM.pdf]

nature research

Corresponding author(s): NCOMMS-18-31618A

Last updated by author(s): Jun 16, 2019

Reporting Summary

Nature Research wishes to improve the reproducibility of the work that we publish. This form provides structure for consistency and transparency in reporting. For further information on Nature Research policies, see Authors & Referees and the Editorial Policy Checklist.

Statistics

For all statistical analyses, confirm that the following items are present in the figure legend, table legend, main text, or Methods section.

n/a	Confirmed	
☐	☒	The exact sample size (*n*) for each experimental group/condition, given as a discrete number and unit of measurement
☐	☒	A statement on whether measurements were taken from distinct samples or whether the same sample was measured repeatedly
☐	☒	The statistical test(s) used AND whether they are one- or two-sided<br>*Only common tests should be described solely by name; describe more complex techniques in the Methods section.*
☒	☐	A description of all covariates tested
☒	☐	A description of any assumptions or corrections, such as tests of normality and adjustment for multiple comparisons
☐	☒	A full description of the statistical parameters including central tendency (e.g. means) or other basic estimates (e.g. regression coefficient) AND variation (e.g. standard deviation) or associated estimates of uncertainty (e.g. confidence intervals)
☐	☒	For null hypothesis testing, the test statistic (e.g. *F*, *t*, *r*) with confidence intervals, effect sizes, degrees of freedom and *P* value noted<br>*Give P values as exact values whenever suitable.*
☒	☐	For Bayesian analysis, information on the choice of priors and Markov chain Monte Carlo settings
☒	☐	For hierarchical and complex designs, identification of the appropriate level for tests and full reporting of outcomes
☐	☒	Estimates of effect sizes (e.g. Cohen's *d*, Pearson's *r*), indicating how they were calculated

Our web collection on statistics for biologists contains articles on many of the points above.

Software and code

Policy information about availability of computer code

Data collection	The RNA-seq data were generated by Illumina Hiseq 4000 platform by Annoroad Gene Technology Co., Ltd. The genomic DNA were sequenced on a HiSeq 2000 sequencer by WuXi NextCODE. Fow cytometry data were collected by BD Fortessa II and cell sorting was performed by BD FACSAria. All images were capture by Nikon Ti-S microscopy.
Data analysis	TopHat v2.0.13 was used for mapping homo sapiens reference genome. Cufflinks v2.2.0 was used to construct, identify,estimate the abundance from TopHat alignment results and analyzed the gene expression. CANOES, was used to detect large exonic deletions and duplications. R 3.3.2 was used as platform for bioinformatics data analysis. Flowjo X was used to analyze the flow cytometry data. GraphPad prism 7 was used for generated the charts and statistical analyses.

For manuscripts utilizing custom algorithms or software that are central to the research but not yet described in published literature, software must be made available to editors/reviewers. We strongly encourage code deposition in a community repository (e.g. GitHub). See the Nature Research guidelines for submitting code & software for further information.

Data

Policy information about availability of data

All manuscripts must include a data availability statement. This statement should provide the following information, where applicable:

- Accession codes, unique identifiers, or web links for publicly available datasets
- A list of figures that have associated raw data
- A description of any restrictions on data availability

The raw RNA-seq data using in this study are available in the NCBI Gene Expression Omnibus (GEO) database under accession number GSE116685.

Field-specific reporting

Please select the one below that is the best fit for your research. If you are not sure, read the appropriate sections before making your selection.

☒ Life sciences ☐ Behavioural & social sciences ☐ Ecological, evolutionary & environmental sciences

For a reference copy of the document with all sections, see nature.com/documents/nr-reporting-summary-flat.pdf

Life sciences study design

All studies must disclose on these points even when the disclosure is negative.

Sample size	The sample sizes for the each experiments were described in the relevant figure legends of the paper
Data exclusions	No data were excluded from the analyses
Replication	All of attempts to replicate experiments were successful
Randomization	Mice used for the biological replication were from the same generation or as little mates, samples were allocated into experimental groups base on the genotype or properties.
Blinding	The investigators were blinded to the genotype of the sample or mouse model, but the phenotype were quantifiable due to the the volume of thymus and staining intensity base on the experience.

Reporting for specific materials, systems and methods

We require information from authors about some types of materials, experimental systems and methods used in many studies. Here, indicate whether each material, system or method listed is relevant to your study. If you are not sure if a list item applies to your research, read the appropriate section before selecting a response.

Materials & experimental systems

n/a	Involved in the study
☐	☒ Antibodies
☐	☒ Eukaryotic cell lines
☒	☐ Palaeontology
☐	☒ Animals and other organisms
☐	☒ Human research participants
☒	☐ Clinical data

Methods

n/a	Involved in the study
☒	☐ ChIP-seq
☐	☒ Flow cytometry
☒	☐ MRI-based neuroimaging

Antibodies

Antibodies used

Mouse Lineage Panel Biotin label BD® Bioscience
Sca-1 D7 eBioscience™
C-Kit 2B8 eBioscience™
CD3e 145-2C11 BD® Bioscience
CD4 RM4-5 BD® Bioscience
CD8 53-6.7 eBioscience™
CD44 IM7 eBioscience™
CD25 PC61.5 eBioscience™
CD45.2 104 eBioscience™
B220 RA3-6B2 eBioscience™
CD19 1D3 BD® Bioscience
IgM II/41 eBioscience™
IL-7R SB/199 eBioscience™
FcγR 93 eBioscience™
TCR-Vb5.1 MR9-4 Biolegend
Streptavidin V450-Streptavidin BD® Bioscience
SETD2 #23486 Cell Signaling Technology
SETD2 #3194 Abclonal
HA-tag #3724s Cell Signaling Technology
H3K36me3 ab9050 Abcam
H3K36me2 ab3594 Abcam
H3K4me3 #9751 Cell Signaling Technology
H3 ab1791 Abcam
RAG1 #3968s Cell Signaling Technology

RAG2 ab133609 Abcam
γH2A.X (p-S139) ab26350 Abcam
ATM ab78 Abcam
ATM (p-S1981) ab81292 Abcam
β-Actin #3700 Cell Signaling Technology

Validation	Mouse Lineage Panel Biotin label BD® Bioscience validation reference PMID: 28218906<br>Sca-1 D7 eBioscience™ validation reference PMID: 21948176<br>C-Kit 2B8 eBioscience™ validation reference PMID: 21948176<br>CD3e 145-2C11 BD® Bioscience validation reference PMID: 28368009<br>CD4 RM4-5 BD® Bioscience validation reference PMID: 11001905<br>CD8 53-6.7 eBioscience™ validation reference PMID: 25485616<br>CD44 IM7 eBioscience™ validation reference PMID: 22673910<br>CD25 PC61.5 eBioscience™ validation reference PMID: 27323847<br>CD45.2 104 eBioscience™ validation reference PMID: 24487275<br>B220 RA3-6B2 eBioscience™ validation reference PMID: 25575242<br>CD19 1D3 BD® Bioscience validation reference PMID: 25575242<br>IgM II/41 eBioscience™ validation based on manufacturer's data sheet<br>IL-7R SB/199 eBioscience™ validation based on manufacturer's data sheet<br>FcγR 93 eBioscience™ validation based on manufacturer's data sheet<br>TCR-Vb5.1 MR9-4 Biolegend validation based on manufacturer's data sheet<br>Streptavidin V450-Streptavidin BD® Bioscience validation reference PMID: 27792766<br>SETD2 #23486 Cell Signaling Technology validation based on manufacturer's data sheet<br>SETD2 #3194 Abclonal validation reference PMID: 28753426<br>HA-tag #3724s Cell Signaling Technology validation reference PMID: 25042806<br>H3K36me3 ab9050 Abcam validation reference PMID: 25693568<br>H3K36me2 ab3594 Abcam validation based on manufacturer's data sheet<br>H3K4me3 #9751 Cell Signaling Technology validation reference PMID: 20385584<br>H3 ab1791 Abcam validation reference PMID: 25370275<br>RAG1 #3968s Cell Signaling Technology validation reference PMID: 25572281<br>RAG2 ab133609 Abcam validation reference PMID: 24614102<br>γH2A.X (p-S139) ab26350 Abcam validation reference PMID: 26374986<br>ATM ab78 Abcam validation reference PMID: 16439685<br>ATM (p-S1981) ab81292 Abcam validation reference PMID: 26344566<br>β-Actin #3700 Cell Signaling Technology validation reference PMID: 25240927

Eukaryotic cell lines

Policy information about cell lines

Cell line source(s)	293T cell line purchased from the Cell bank of China Academy of Science
Authentication	The authentication of 293T cell line was perform by the Cell bank of China Academy of Science
Mycoplasma contamination	Cell lines were tested for mycoplasma presence using the mycoplasma test kit.
Commonly misidentified lines<br>(See ICLAC register)	No commonly misidentified cell lines were used.

Animals and other organisms

Policy information about studies involving animals; ARRIVE guidelines recommended for reporting animal research

Laboratory animals	C57BL/6 mice,both male and female, were used in this study, and described in the paper for detailes.
Wild animals	The study did not involved wild animals.
Field-collected samples	The study did not involve the samples collected from the field
Ethics oversight	All mice were maintained and bred in the pathogen-free facility at Renji Hospital. Mouse experimental protocols were approved by the Renji Hospital Animal Care and Use Committee.

Note that full information on the approval of the study protocol must also be provided in the manuscript.

Human research participants

Policy information about studies involving human research participants

Population characteristics	These patients were from a nonconsanguineous Chinese family with healthy parents and without a family history of specific diseases. All the characteristics are listed in Supplementary Table 6.

Recruitment	All patients information and samples were collected based on clinical requirements for diagnosis.
Ethics oversight	This study was approved by the ethics committee of the Children's Hospital of Fudan University. Written informed consent was obtained from the parents of all patients. The study was conducted in accordance with the approved guidelines.

Note that full information on the approval of the study protocol must also be provided in the manuscript.

Flow Cytometry

Plots

Confirm that:

☒ The axis labels state the marker and fluorochrome used (e.g. CD4-FITC).

☒ The axis scales are clearly visible. Include numbers along axes only for bottom left plot of group (a 'group' is an analysis of identical markers).

☒ All plots are contour plots with outliers or pseudocolor plots.

☒ A numerical value for number of cells or percentage (with statistics) is provided.

Methodology

Sample preparation	The freshed mice samples were disscociated into single cell suspensions and filtered with 40 μm cell strainer, lysated the red blood cell and washed three times with ice cold PBS and incubated with primary antibodies and followed second antibodies, then collected cells via centrifuge and resuspended cells with PBS and analysis.
Instrument	Fow cytometry data were collected by BD Fortessa II and cell sorting was performed by BD FACSAria.
Software	FlowJo X
Cell population abundance	The relevant cell populations were at lest collected 2000 using the BD FACSAria cell sorting platform, and confirmed the purity of sorted cell by re-analyzed via BD Fortessa II.
Gating strategy	The gating strategies were described in Supplementary figure 10

☒ Tick this box to confirm that a figure exemplifying the gating strategy is provided in the Supplementary Information.
